# Confirmation of endoscopic injection sclerotherapy by mucosal whitening in the treatment of hemorrhage of refractory recurrent tiny esophageal varices

**DOI:** 10.1055/a-2578-2909

**Published:** 2025-05-06

**Authors:** Kazunori Nagashima, Shintaro Yamaguchi, Ryuichi Maki, Fumihiko Urushibara, Tsunehiro Suzuki, Toshimitu Murohisa, Atsushi Irisawa

**Affiliations:** 1 Department of Gastroenterology, Dokkyo Medical University School of Medicine, Shimotsuga, Japan; 2 Japanese Red Cross Ashikaga Hospital, Ashikaga, Japan

Endoscopic injection sclerotherapy (EIS) is particularly useful for refractory recurrent scarring small varices with red color sign for which endoscopic variceal ligation (EVL) is not feasible. In such cases, intravariceal injection can be confirmed by observing whitening of the mucosa (because of local ischemia) after injection of the sclerosing agent. This report is the first video case confirming that the mucosa around tiny varices whitens during intravariceal EIS.

This video presents a typical case ( Video 1 ). A 75-year-old man with portal vein thrombosis visited our hospital, complaining of hematemesis. He had undergone EVL for esophageal varices in the past. Emergency endoscopy revealed hemorrhagic small esophageal varices with multiple scars caused by EVL ( Fig. 1 , Fig. 2 ). After inflating a balloon attached to the endoscope tip, the varices were punctured using a 25-gauge needle (Varixer; TOP Corp., Tokyo, Japan). A sclerosant, ethanolamine oleate, was injected into the small varices with contrast medium. Fluoroscopy showed the sclerosant injected into the varix toward the blood supply route ( Fig. 3 ). The mucosa around the sclerosant-injected varices became white during ethanolamine oleate injection ( Fig. 4 ). The treatment was completed and hemostasis was achieved ( Fig. 5 ). No early or late adverse event was related to this procedure.

Confirmation of sclerosant injection by mucosal whitening.Video 1

**Fig. 1:**
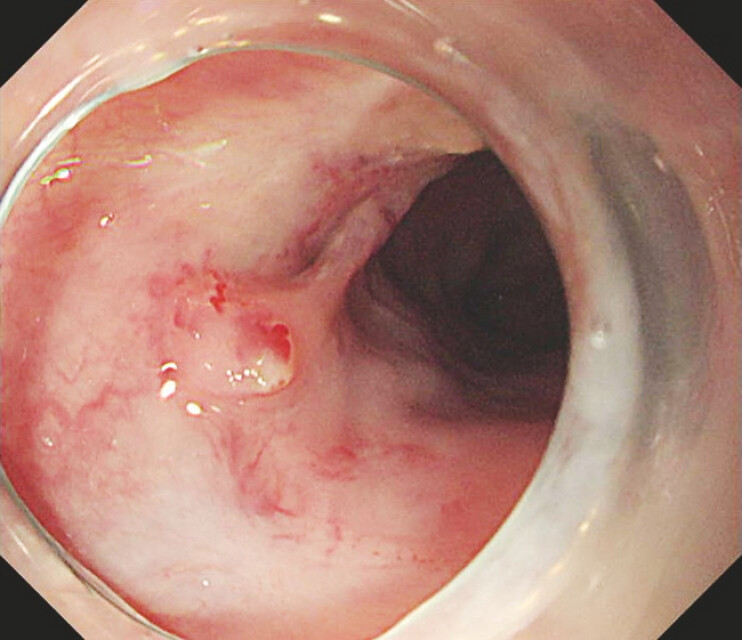
Recurrent tiny varices were located in the scarred mucosa.

**Fig. 2:**
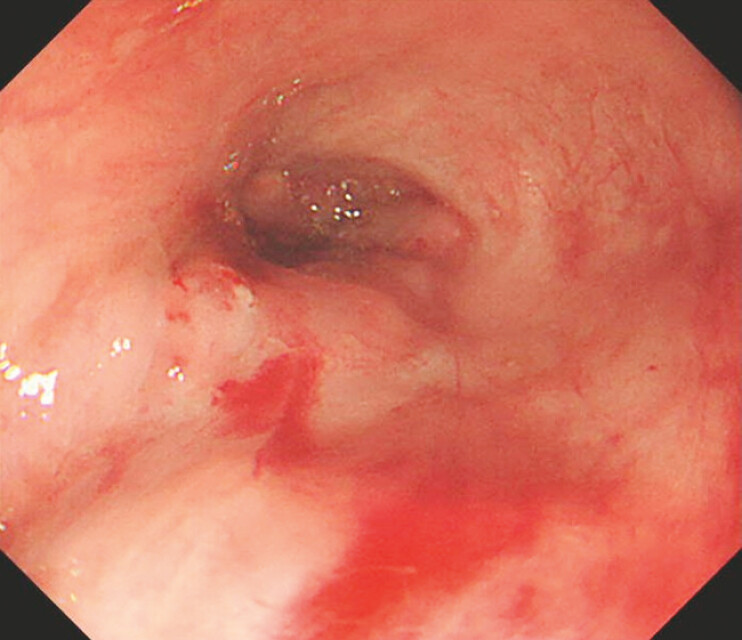
Varices at the 7 o’clock position were bleeding.

**Fig. 3:**
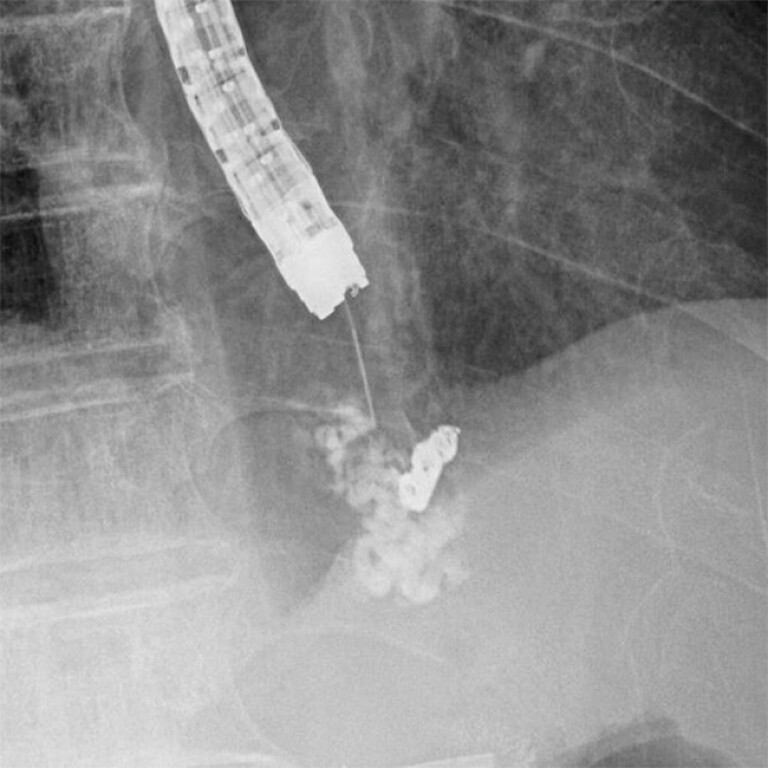
Sclerosant was injected toward the blood supply route.

**Fig. 4:**
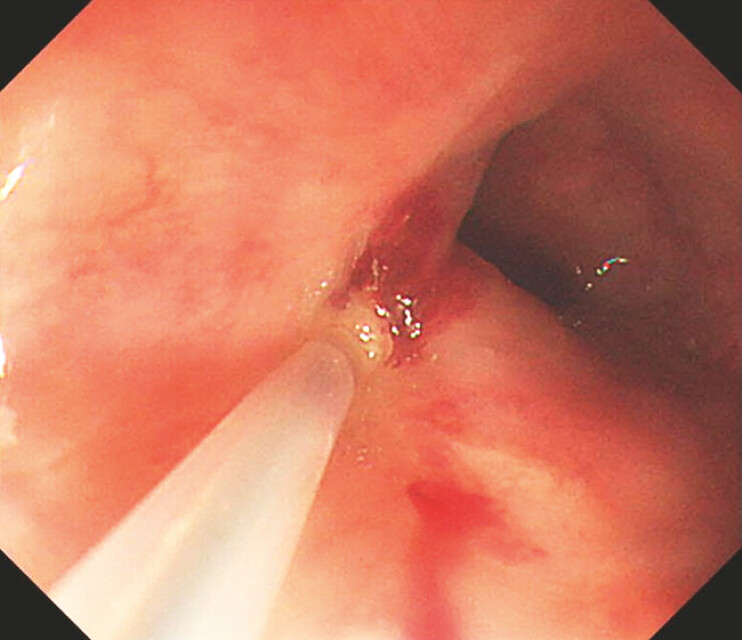
The mucosa around the varices turned white during intravascular injection.

**Fig. 5:**
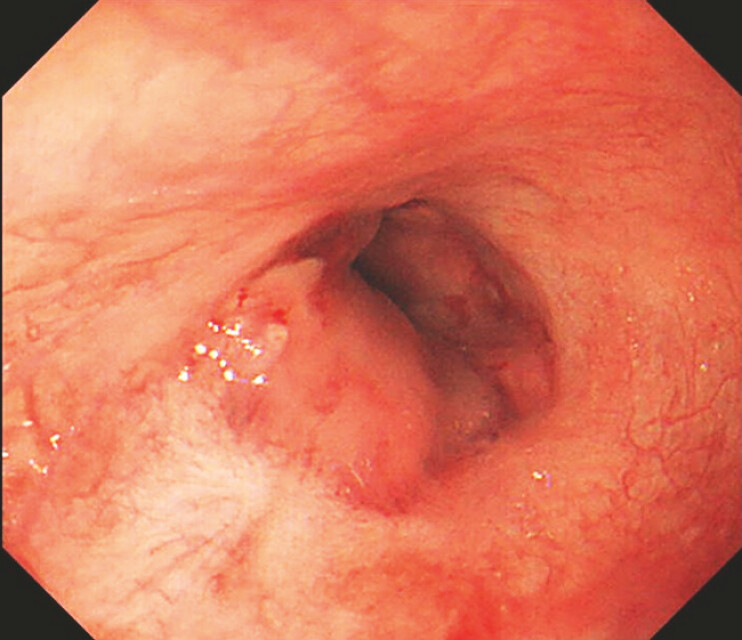
The varices were hemostatic.

Varices with red color sign are at risk of bleeding and should be treated, even if they are recurrent or small 1 . For recurrence or scarring small varices after endoscopic treatment as described above, EVL is difficult to perform, and EIS is more effective 2 3 . It is important to perform intravascular injection and thoroughly embolize the blood supply route during EIS. Although limited to extremely small varices, if the mucosa becomes white after sclerosant injection, as in this case, it confirms that intravascular injection has been performed reliably, even if it cannot be confirmed using fluoroscopy. This finding indicates reliable embolization effects on small varices.

Endoscopy_UCTN_Code_TTT_1AO_2AD

## Notes

Endoscopy E-Videos https://eref.thieme.de/e-videos
*E-Videos* is an open access online section of the journal *Endoscopy* , reporting on interesting cases and new techniques in gastroenterological endoscopy. All papers include a high-quality video and are published with a Creative Commons CC-BY license. Endoscopy E-Videos qualify for HINARI discounts and waivers and eligibility is automatically checked during the submission process. We grant 100% waivers to articles whose corresponding authors are based in Group A countries and 50% waivers to those who are based in Group B countries as classified by Research4Life (see: https://www.research4life.org/access/eligibility/ ). This section has its own submission website at https://mc.manuscriptcentral.com/e-videos .
